# Effect of Kinesio tape and Compression sleeves on delayed onset of muscle soreness: a single-blinded randomized controlled trial

**DOI:** 10.1186/s12891-023-06499-3

**Published:** 2023-05-17

**Authors:** Xiali Xue, Yuerong Hao, Xinwei Yang, Chaoyang Zhang, Jie Xu, Xiaolei Wu, Zhongyi Deng, Ning Li

**Affiliations:** 1 Institute of Sports Medicine and Health, Chengdu Sport University, Chengdu, 610041 Sichuan China; 2 School of Physical Education, Qingdao University, Qingdao, 266071 Shandong China; 3 Department of Sports Medicine, Sichuan Province Orthopedic Hospital, Chengdu, 610041 Sichuan China; 4 Department of Rehabilitation Medicine, The Third Affiliated Hospital, Sun Yat-Sen University, Guangzhou, 510630 Guangdong China

**Keywords:** Delayed onset muscle soreness (DOMS), Kinesio tape, Isokinetic strength, Interleukin-6, Fatigue

## Abstract

**Background:**

Both Kinesio Tape (KT) and Compression Sleeves (CS) can relieve Delayed Onset Muscle Soreness (DOMS) to a certain extent, but there is no study report on the difference in the effectiveness of the KT and CS whether the effect is better when used at the same time. The purpose of this study was to compare the effects of KT and CS on the recovery of muscle soreness, isokinetic strength, and body fatigue after DOMS.

**Methods:**

In this single-blinded randomized controlled trial, 32 participants aged 18 to 24 years were randomly divided into Control group (CG), Compression Sleeves group (CSG), Kinesio Tape group (KTG), Compression Sleeves and Kinesio Tape group (CSKTG), between October 2021 and January 2022. KTG uses Kinesio Tape, CSG wears Compression Sleeves, and CSKTG uses both Compression Sleeves and Kinesio Tape. Outcomes were performed at five-time points (baseline, 0 h, 24 h, 48 h, 72 h), Primary outcome was pain level Visual Analogue Scale (VAS), and Secondary outcomes were Interleukin 6, Peak Torque/Body Weight, Work Fatigue. Statistical analyses were performed using the repeated measures analysis of variance method. Setting: Laboratory.

**Results:**

After the intervention, VAS reached the highest at 24 h after exercise-induced muscle soreness, while the KTG and CSG at each time point were less than CG, and the scores of CSKTG at 24 h and 48 h were less than those of KTG and CSG in the same period (P < 0.05). Interleukin 6, at 24 h, CSKTG is lower than KTG 0.71(*95%CI:* 0.43 to 1.86) and CG 1.68(*95%CI:* 0.06 to 3.29). Peak Torque/Body Weight, at 24 h, CG was lower than CSKTG 0.99(*95%CI:* 0.42 to 1.56), KTG 0.94(*95%CI:* 0.37 to 1.52), and CSG 0.72(*95%CI:* 0.14 to 1.29); at 72 h, CG was lower than CSKTG 0.65(*95%CI:* 0.13 to 1.17) and KTG 0.58(*95%CI:* 0.06 to 1.10). Work Fatigue, at 24 h, CG was lower than KTG 0.10(*95%CI:* 0.02 to 1.78) and CSKTG 0.01(*95%CI:* -0.07 to 0.09). At 48 h, CG was lower than KTG 0.10(*95%CI:* 0.13 to 1.17) and CSKTG 0.11(*95%CI:* 0.03 to 0.18).

**Conclusions:**

Kinesio Tape can significantly reduce DOMS pain, and Kinesio Tape has a better recovery effect on Delayed Onset Muscle Soreness than Compression Sleeves. Kinesio Tape combined with Compression Sleeves is helpful to alleviate the Delayed Onset Muscle Soreness pain, speeding up the recovery of muscle strength, and shortening the recovery time after Delayed Onset Muscle Soreness.

**Trial registration:**

Registration number: This study was also registered on 11/10/2021, at the Chinese Clinical Trial Registry (ChiCTR2100051973).

## Introduction

Delayed onset muscle soreness (DOMS) is typical ultrastructural muscle damage, which usually occurs after unaccustomed or high-intensity eccentric muscle contraction [1]. The symptoms strengthen in 12-24 h after exercise, reach a peak within 24-72 h, and gradually reduce within 5–7 days after exercise [2, 3]. DOMS can result from unaccustomed eccentric training. DOMS usually occurs after an increase in the intensity or volume of training or when the schedule of exercises is altered or a new one is implemented. Exercise intensity and duration are also important factors in clinical symptoms of DOMS including reduced muscle strength, restricted movement, pain, mild tenderness, stiffness, swelling, and adjacent joint dysfunction [4, 5].

Although DOMS is considered to be a mild injury, it is one of the most common causes of impaired exercise performance, and it has a high incidence [6]. Competition and training can induce repeated eccentric contractions, which may lead to muscle damage (i.e. destruction of structural proteins in muscle fibers and/or connective tissue), subsequent tissue inflammation, increased DOMS, and perceived fatigue. The observed changes in the blood concentration of interleukin-6 (IL-6), the inflammatory biomarker of muscle injury after exercise, is related to the occurrence of DOMS and skeletal muscle recovery [7]. In the past decades, many hypotheses have been proposed to explain the etiology of DOMS. Although the exact pathophysiological pathway is unclear, the main mechanism is the ultrastructural damage of muscle cells caused by eccentric exercise, which leads to further protein degradation, apoptosis, and local inflammatory response. The development of clinical symptoms is usually delayed due to the complex sequence of local and systemic physiological reactions [8]. Therefore, it is particularly important to better alleviate muscle pain, swelling, limited activity, and adjacent joint dysfunction after exercise, and prevent DOMS. Previous research has proposed many interventions in an attempt to mitigate the side effects associated with this type of exercise. Such as neuromuscular electrical stimulation [9], cold therapy [10], foam rolling [11], massage [12], etc. However, there is still no gold standard for the treatment of DOMS.

Kinesio tape (KT) is an ultra-thin breathable tape with excellent elasticity, which is used to treat sports injuries and many other diseases. It was first invented by Dr. Kenso Kase of Japan in 1973. Its name comes from kinesiology, which takes the prefix of the English word as the name “Kinesio tape”. At present, it is widely used in the fields of skeletal muscle limitation and sports injury [13, 14]. The main function of KT is to support injured muscles and joints, enhance muscle strength, improve muscle function, inhibit muscle tension, and help mitigate pain by improving skin and blood, and lymph flow [15]. It can mitigate abnormal muscle tension, relax soft tissue or promote soft tissue functional activities, and help restore fascia and muscle function [16–19].

With the rapid development of science and technology and the increasingly mature sports training level, high-tech sports equipment plays an important role in the field of leisure and public fitness as well as intense competition. In recent years, compression equipment (Pressure suits, pressure socks, tights, compression sleeves, etc.) with its unique technology, wide application, and its unique effect, has gradually been favored by domestic and foreign athletes and fitness [20, 21]. Recently years, as a form of compression equipment, Compression Sleeves (CS) have been a hot spot in the research of sports medicine and biomechanics. CS mainly produces an external pressure gradient, reduces swelling space, improves venous reflux, reduces venous pool, and enhances metabolite clearance, which may be due to enhancing muscle pump function and reducing secondary inflammatory response [22–24]. CS is becoming increasingly popular among athletes because of its ability to improve athletic performance, reduce symptoms and risk of injury, and facilitate athletic recovery [21, 25]. Some studies have reported that CS can promote the recovery of DOMS, especially muscle pain, injury, and inflammation after exercise, mainly related to CS compression [23, 26, 27]. Diego et al. found that when football players wear full-leg compression suits or compression sleeves, inflammation and perceptual reactions indicate that oppression can be an interesting strategy to promote physiological and psychological recovery [28]. A meta-analysis by Freddy et al. showed that compression garments most effectively promoted recovery of the resistance movement, especially 24 h after exercise [29]. Goto et al. conducted 70% 1RM resistance training for 9 well-trained men and found that pressure clothing can promote strength recovery, and reduce muscle soreness and tissue edema [30]. Kraemer et al. conducted two groups of 50 times/group of passive arm bending exercises (eccentric stage) for 20 healthy women without resistance training experience. They found that compression sleeves can effectively reduce the serum creatine kinase concentration after eccentric training, reduce muscle soreness and promote strength recovery [31]. At present, although the knowledge of the physiological and biochemical effects of wearing CS is not enough, the positive ideas related to tight-fitting equipment have led to an increase in the use of CS in sports.

From the literature review, we found that KT and CS have similar mechanisms of action, which have great significance in reducing DOMS and avoiding further damage. However, there is not a simple and effective method to prevent or eliminate DOMS at present. Although the above two methods can alleviate DOMS to a certain extent, there is no literature to report the differences between the two methods and how it is better to use the two methods at the same time. Combined with the occurrence characteristics of DOMS, this study designed an innovative experiment and analyzed the treatment effect based on quantitative data, to explore which of KT and CS after DOMS can better promote the recovery of muscle soreness, and physical function (Isokinetic muscle strength, Fatigue index).

### Study aim and hypothesis

The aim was to compare the effects of KT and CS on the recovery of muscle soreness, isokinetic strength, and body fatigue after DOMS. At the same time, it is hoped that this study can provide guidance and help for clinicians and researchers to prevent DOMS during exercise.

#### H1

Compared with the control group, the combined use of KT and CS is better than the single use of KT and CS in preventing or reducing DOMS.

#### H2

Compared with the control group, the combination of KT and CS could reduce DOMS by reducing inflammation and factors such as muscle damage and pain.

## Methods

### Study design

This study was conducted between October 2021 and December 2021. This was a single-blinded randomized controlled study. The study protocol follows the Consolidated Standards of Reporting Trials (CONSORT) statement on randomized trials of non-pharmacological treatment [32]. This study was approved by the Ethics Committee of the Chengdu Sport University (No. 2021–48) and the participants provided informed consent. This study was also registered on 11/10/2021, at the Chinese Clinical Trial Registry (ChiCTR2100051973), http://www.chictr.org.cn/showproj.aspx?proj=65929.

### Sample size

There is no relevant comparative experimental research before, so we did not use data from the literature to calculate the sample size. Based on the previous pilot experiment, the main outcome measurement index VAS was used to estimate the sample size, assuming that the effect size was 0.4, using the G*Power3.1 sample size calculation software, and following the calculation method of the F test repeated measurement analysis of variance between groups, set the group as 4 groups, the number of repeated measurements as 5 times, set the effect size as 0.25, the significance level *P* = 0.05, POWER = 0.95. Thus, the total sample size was calculated to be 32 participants. To compensate for the 10% dropout rate, a total of 36 participants was recruited.

### Randomization and blinding

All participants meeting inclusion criteria were selected and allocated into four groups by using the computer-generated random sequence (simple randomization) and set in a sealed envelope before baseline assessment. The therapist will communicate the grouping assigned to the participants. Due to the nature of the intervention, it is not possible to blind either the therapist or the participant. Evaluators unaware of the group assignment will perform outcome measurements at all time points. Data entry and statistical analysis will be performed without knowledge of the interventions. The random plan was only known to the project leader. Consistent with the Declaration of Helsinki, all participants provided signed written informed consent.

### Participants

Inclusion criteria: Male, aged 18–24, with a history of irregular exercise; Do not participate in other sports activities 2 weeks before and during the test; Height range: 165 cm-180 cm, weight range: 60 kg-80 kg; Healthy, free from cardiovascular diseases, muscle damage and pain, and neurological diseases; Voluntarily participated in the study without any discomfort and sign informed consent.

Exclusion criteria were: Participants who are taking drugs; Participants with musculoskeletal injury of lower limbs within 6 months; The presence of any medical condition which has been recommended by the doctor to prevent participation in sports; Participants who have muscle aches or chronic pain symptoms; Loss of sensation or numbness of upper and lower limbs; Participants with chest pain, dyspnea, or feeling uncomfortable during exercise; Any history of cardiovascular disease, lung disease, heart disease, cardiopulmonary insufficiency, and other vital organ insufficiencies; Major diseases or congenital diseases.

### Group allocation and assessment

**Fig. 1:**
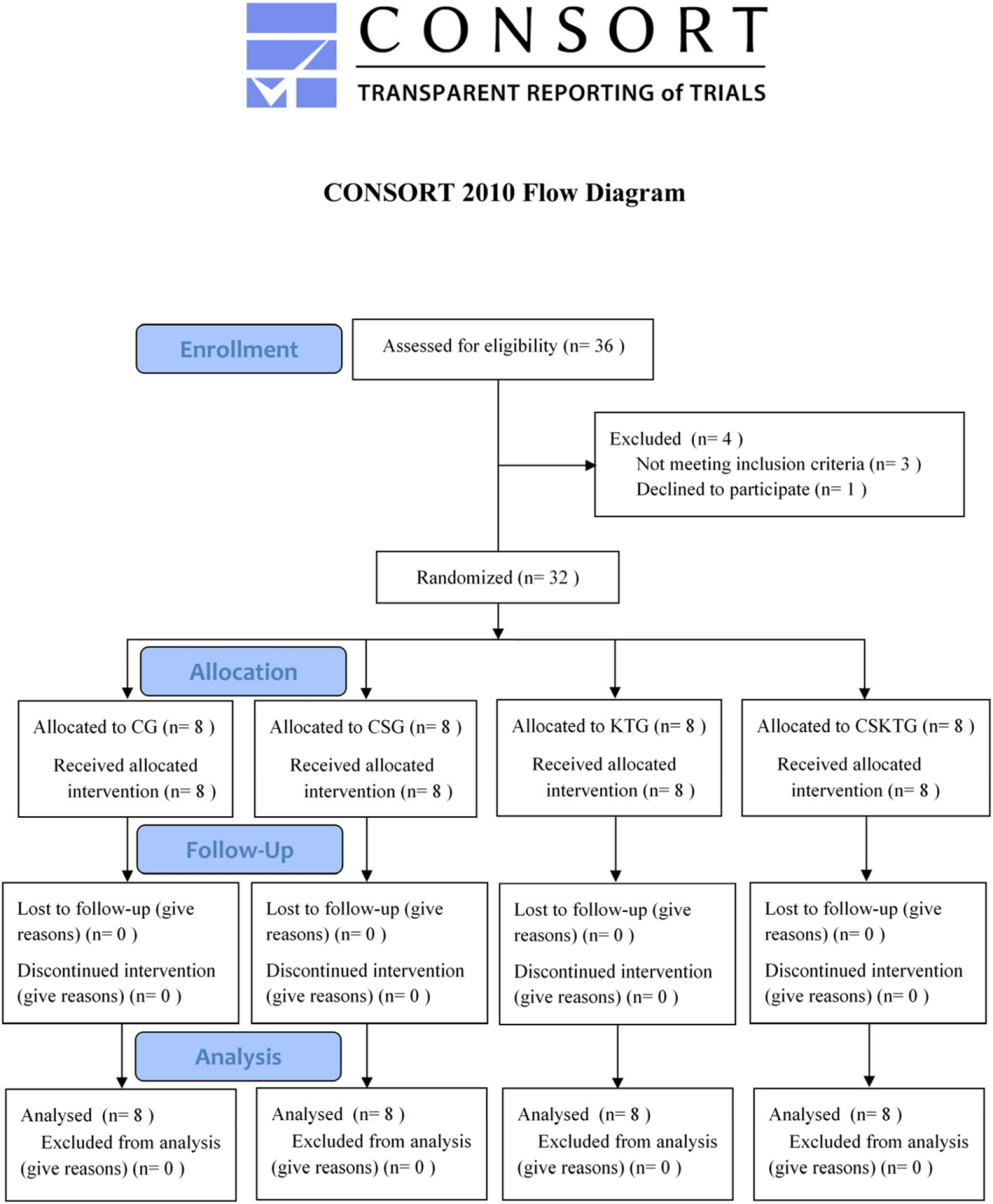
Flowchart of the study. Participants’ flow through the study based on the Consolidated Standards of Reporting Trials (CONSORT) flow diagram. A total of 36 participants were recruited in this study, of which 1 refused to participate in this experimental study, 3 did not meet the inclusion criteria, and finally, 32 participants were included

All participants were equally divided into the Control group (CG), the Compression sleeves group (CSG), the Kinesio tape group (KTG), and the Compression sleeves and Kinesio tape group (CSKTG) by a random digital table with each card placed in a brown opaque envelope. Each group had 8 participants, and participants were assessed in a separate room before and after the intervention. The study flowchart is shown in Fig. 1.

### Intervention methods and measures

All participants perform an exercise-induced muscle damage model after the baseline measurement. Combined with the previous exercise-induced muscle damage methods, participants will continuously perform 5 groups of 20 drop jumps from a 0.6 m high box, with an interval of 10 s. The rest time between the two groups was 2 min [33]. The participants showed muscle soreness, stiffness, decreased muscle strength, and muscle dysfunction 24 h after the modeling was used as the basis for judging the success of the exercise modeling. In the process of modeling, the control group did not carry out other additional interventions except modeling, CSG wore Compression sleeves, KTG applied Kinesio tape, and CSKTG used both Compression sleeves and Kinesio tape. The intervention site is the laboratory of the Institute of Sports Medicine and Health, Chengdu Sport University. All results will be collected at baseline, immediately after modeling, 24 h, 48 h, and 72 h. The IL-6 index was only tested 48 h after modeling.

**Fig. 2:**
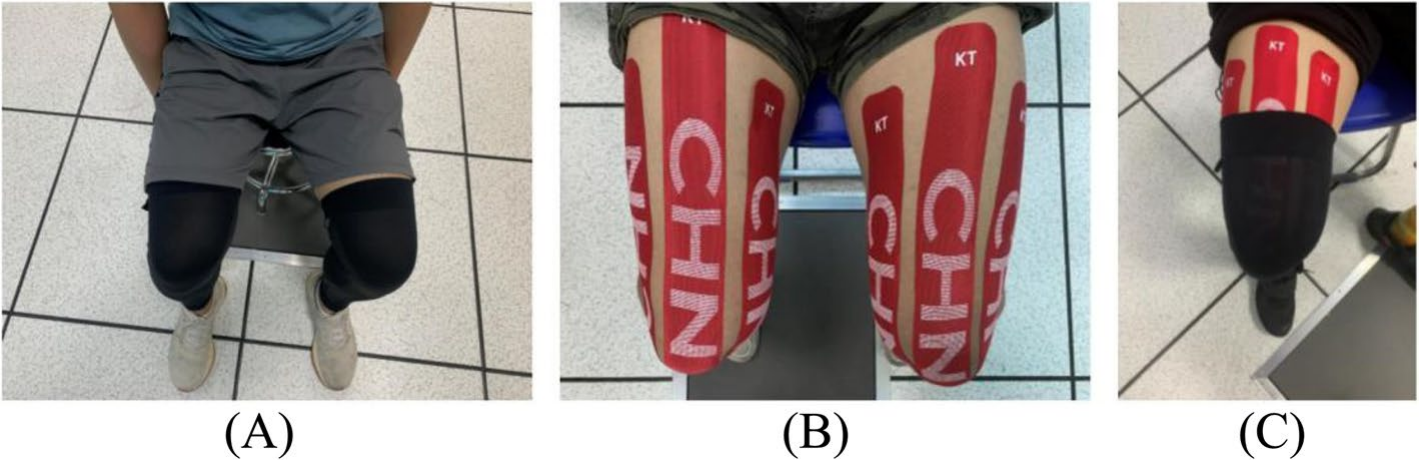
Interventions for the groups in the experimental group. The wearing of Compression sleeves is shown in (**A**); The sticking Kinesio tape is shown in (**B**); Compression sleeves and Kinesio tape group stick stuck according to the Kinesio tape group's standard, and then put on tight equipment for the test. Specific wear is shown in (**C**)

**Fig. 3:**
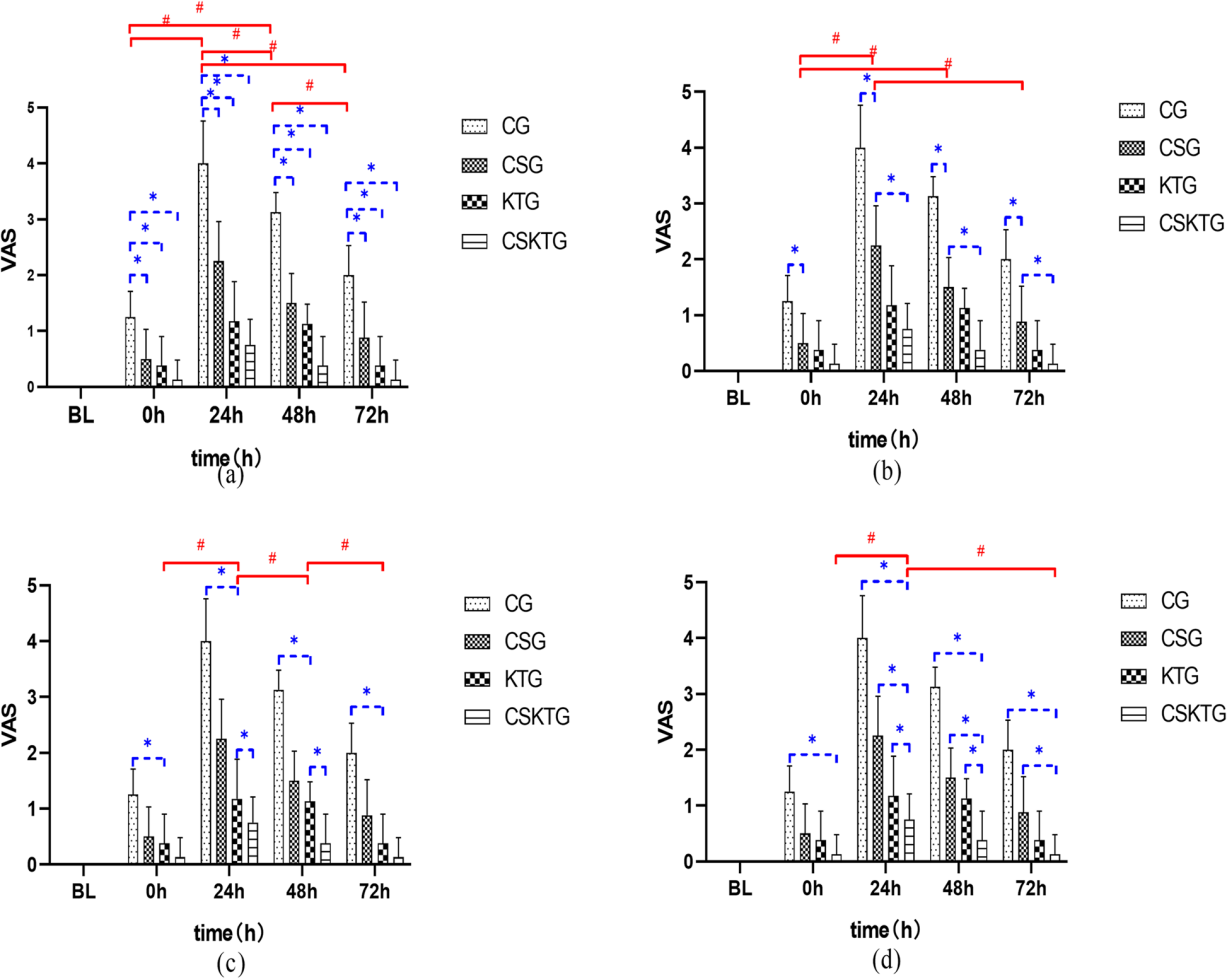
Comparison of VAS value between-groups and within-groups. (**a**), (**b**), (**c**) and (**d**) were the comparison of VAS value between-groups and within-groups in CG, CSG, KTG and CSKTG groups. *: Pairwise comparison between-groups *P*’ < 0.05. ^#^: Pairwise comparison within-groups *P*’ < 0.05

**Fig. 4:**
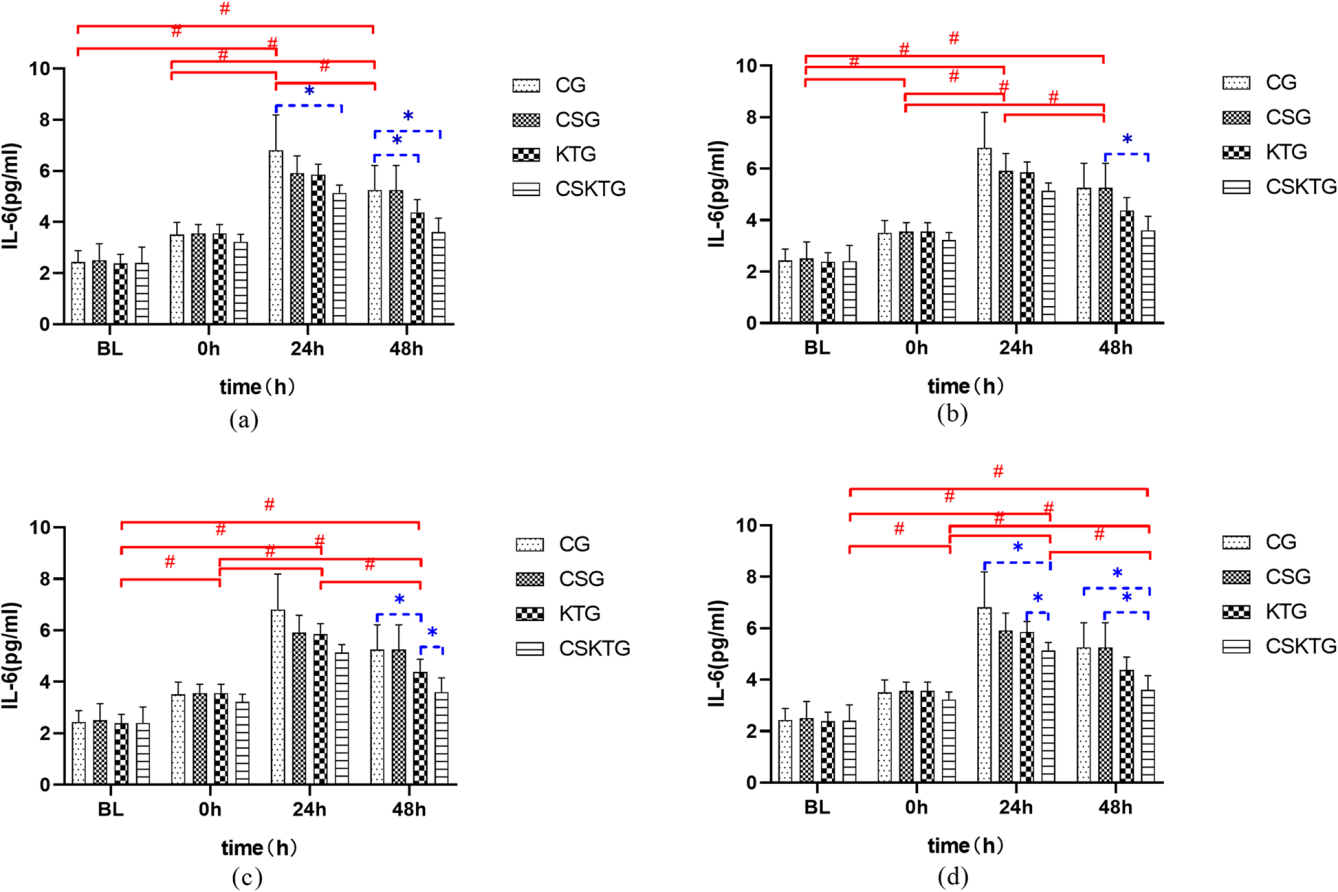
Comparison of IL-6 value between-groups and within-groups. (**a**), (**b**), (**c**) and (**d**) were the comparison of IL-6 value between-groups and within-groups in CG, CSG, KTG and CSKTG groups. *: Pairwise comparison between-groups *P*’ < 0.05. ^#^: Pairwise comparison within-groups *P*’ < 0.05

The specific intervention measures of the four groups were (1) The Control group no other intervention except modeling; (2) The Compression sleeves group wears the selected brand of lower limb Compression sleeves (Brand: LP, Model: LP-272Z, Origin: Taiwan Province, China) before modeling, which was suitable for people with a dimension of the knee joint of 36.0–40.0 cm and a dimension of the middle leg of 35.0–38.0 cm. To avoid affecting the test results due to the difference in the leg circumference of the subjects, the dimension of the knee joint and calf circumference of the subjects were measured before the start of the test to ensure the successful completion of the test. The wearing of Compression sleeves is shown in Fig. 2 (A); (3) The participants of the Kinesio tape group took the sitting position, kept the knee joints of both lower limbs bent at 90°, and stuck on the skin surface of the rectus femoris, lateral femoral muscle, and medial femoral muscle of both thighs. Kinesio tape was 5 cm × 25 cm, manufactured by KT (USA). The pasting shape was “I”, with the natural tension (110% of the original length). The classic Kase relaxation pasting method and facilitation technology are adopted to paste from the starting point to the endpoint [34]. Cut three KT with a width of 5 cm and a length of 20 cm. The starting point of the patch was the tibial tuberosity. The three Kinesio tapes span the knee joint upward and end at the distal end of the medial femoral muscle, rectus femoris muscle, and lateral femoral muscle of the thigh respectively. There was no tension at both ends of the patch. The sticking demonstration is shown in Fig. 2 (B); (4) Compression sleeves and Kinesio tape group stuck according to the Kinesio tape group's standard, and then put on tight equipment for the test. Specific wear is shown in Fig. 2 (C).

### Outcome measures

The outcomes were measured baseline, immediately after modeling, 24 h, 48 h, and 72 h. Blood outcomes will not be tested after 48 h of modeling. The outcome measures will be as follows.

The primary outcome was muscle pain intensity as measured by the Visual Analogue Scale (VAS). Each VAS will consist of a 100 mm horizontal line with the text ‘no pain’ at the 0 mm mark and ‘worst pain imaginable’ at the 100 mm mark. Participants marked pain levels on the lines according to how they felt.

The secondary outcomes were: (1) The biomarkers of peripheral muscle fatigue were used to deeply understand the fatigue mechanism during exercise to detect abnormal fatigue or defective metabolic pathways. Inflammatory factor Interleukin 6 (IL-6) is one of the most commonly used biochemical outcomes to judge muscle fatigue [35]. Blood biochemical outcomes: IL-6, IL-6 is a cytokine, a protein produced by immune cells acting on other cells, which helps to regulate and promote an immune response. It can also stimulate the production of acutely related reactants, and increase the blood concentration in case of inflammation or tissue injury [36, 37]. The IL-6 kit was purchased from Lilai Biotechnology Co., Ltd., and 3 ml of fasting venous blood was taken from the subject, placed for more than 30 min, and then placed in a multi-tube rack automatic centrifuge at 2500 rpm for 7 min. Plasma was extracted with a pipette, divided into EP tubes and numbered, and stored in a -70 °C freezer for testing, and enzyme-linked immunosorbent assay (ELISA) was used to detect the concentration of IL-6 in the plasma of participants. (2) Isokinetic muscle strength test of the knee joint: The Con-Trex isokinetic muscle strength test system made in Germany was used to test the subjects' knee joint for 60°/s extension 20 times, in which the relative peak torque index was the average value of the first five data. The subjects took the sitting position and fixed the trunk and hip joints with wide straps. The knee extension mode was selected, and the joint range of motion was 85°. Muscle strength outcome: Peak Torque to body weight ratio (PT/BW); Work Fatigue (WF): this index is the ratio of the last 1/3 of work done for the first 1/3 of work done in the isokinetic muscle strength test. The closer the WF value is to 1, the better the strength and endurance level of the joint muscle group. At the same time, we will also evaluate the safety of KT and CS.

### Statistical analysis

The data were analyzed by SPSS 22.0 software (IBM Corporation, USA). The general count data of the four groups were compared by one-way ANOVA. VAS, IL-6, PT/BW, and WF were all continuous variables, and the analysis of student residuals and Shapiro Wilk test accorded with the test hypothesis. Therefore, the repeated measures analysis of variance method was used to statistically analyze the data in each group before and immediately after modeling, 24 h, 48 h, and 72 h, and evaluate the time and interaction between groups, as well as the main utility of time and groups. After Mauchly's spherical test of the data, the results that do not meet the spherical hypothesis were corrected by epsilon. When the intervention measures interact with time factors, the separate effects of time and groups need to be analyzed respectively; The group independent effect was compared with the index differences between the groups at five different time points by multivariate analysis of variance, and the case of uneven variance after Levene's homogeneity test was analyzed by Welch analysis of variance; Single-factor repeated measures analysis of variance was used to analyze the differences of time factors in different groups, and Bonferroni method was used for pairwise comparison. When there is no interaction between intervention measures and time factors, the main effects of time and groups need to be analyzed and further compared. The significance level was *P* < 0.05.

## Results

### Participants Information

A total of 36 participants was recruited in this study, of which 1 refused to participate in this experimental study, 3 did not meet the inclusion criteria, and finally, 32 participants were included. All 32 participants completed the intervention and evaluation of the results in this experiment. The basic information of the participants in the trial is shown in Table 1, Shapiro Wilk test showed that the data in each group obeyed normal distribution (*P* > 0.05), there was no significant difference between the four groups in age, height, weight, dimension of the knee joint, and the dimension of the middle leg. 32 healthy non-sports major male college students with no regular exercise training were recruited from the Chengdu Sport University.

**Table 1:** Comparisons of the demographic features of the participants at baseline

	CG	CSG	KTG	CSKTG	*P*
	Mean ± SD	Mean ± SD	Mean ± SD	Mean ± SD	
Age (years)	19.75 ± 1.75	19.75 ± 1.67	19.50 ± 1.07	20.00 ± 2.00	0.055
Height (cm)	173.38 ± 6.14	173.75 ± 5.88	173.38 ± 4.27	173.50 ± 2.98	0.440
Weight (kg)	67.88 ± 6.79	66.88 ± 5.08	67.75 ± 8.07	67.00 ± 4.47	0.310
Dimension of the knee joint (cm)	37.84 ± 1.56	37.56 ± 1.02	38.16 ± 1.36	37.83 ± 1.21	0.211
Dimension of the middle leg (cm)	36.70 ± 1.37	37.24 ± 0.93	37.03 ± 1.04	36.84 ± 1.14	0.590

### Safety assessment

CS, KT, and CSKT groups showed no adverse reactions such as skin allergic reaction, skin surface strain, skin ligature, and compression discomfort.

### VAS value

#### The overall analysis of the VAS value

Shapiro Wilk test showed that the data in each group obeyed normal distribution (*P* > 0.05). There was a significant difference in the group's main effect F (3,21) = 60.667, *p* = 0.000, the group had a significant impact on the overall VAS value; The main effect of the time factor on the VAS value of each group was statistically significant F (4,28) = 126.664, *p* = 0.000, the overall intervention time had a significant impact on VAS value. Due to the influence of group factors on VAS value being statistically significant, the pairwise comparison showed *P* < 0.001 compared with CG and CSG; Compared with KTG, *P* = 0.001; CG compared with CSKTG, *P* < 0.001; Compared with CSKTG, *P* = 0.002. Due to the statistical significance of the influence of the time factor on VAS value, the pairwise comparison showed that BL compared with 0 h, *P* < 0.001; BL compared with 24 h after the intervention, *P* < 0.001; BL compared with 48 h after the intervention, *P* < 0.001; BL compared with 72 h after the intervention, *P* = 0.002; Compared with 24 h after the intervention, *P* < 0.001; Compared with 48 h after the intervention, *P* < 0.001; 24 h after intervention and 48 h after the intervention, *P* = 0.040; Compared with 24 h after intervention and 72 h after the intervention, *P* < 0.001; 48 h after the intervention compared with 72 h after the intervention, *P* = 0.001 (Table 2).

**Table 2:** Overall analysis of VAS, IL-6, PT/BW, WF at different time points in the four groups

	Time	VAS ($\overline{x }\pm s$)		IL-6($\overline{x }\pm s$)		PT/BW($\overline{x }\pm s$)		WF($\overline{x }\pm s$)	
CG	BL	0 ± 0		2.44 ± 0.44		2.90 ± 0.23		0.88 ± 0.05	
	0 h	1.25 ± 0.46		3.51 ± 0.48		2.35 ± 0.46		0.80 ± 0.03	
	24 h	4.00 ± 0.76		6.81 ± 1.38		1.74 ± 0.52		0.70 ± 0.06	
	48 h	3.13 ± 0.35		5.25 ± 0.96		2.15 ± 0.55		0.73 ± 0.05	
	72 h	2.00 ± 0.53		-		2.34 ± 0.48		0.76 ± 0.04	
CSG	BL	0 ± 0		2.51 ± 0.64		2.51 ± 0.64		0.87 ± 0.06	
	0 h	0.50 ± 0.53		3.56 ± 0.34		3.00 ± 0.16		0.81 ± 0.06	
	24 h	2.25 ± 0.71		5.91 ± 0.68		2.57 ± 0.41		0.75 ± 0.06	
	48 h	1.50 ± 0.53		5.25 ± 0.96		2.68 ± 0.32		0.80 ± 0.06	
	72 h	0.88 ± 0.64		-		2.83 ± 0.19		0.82 ± 0.06	
KTG	BL	0 ± 0		2.39 ± 0.35		3.01 ± 0.30		0.86 ± 0.07	
	0 h	0.38 ± 0.52		3.56 ± 0.34		2.72 ± 0.36		0.83 ± 0.06	
	24 h	1.75 ± 0.71		5.85 ± 0.41		2.69 ± 0.36		0.80 ± 0.06	
	48 h	1.13 ± 0.35		4.38 ± 0.50		2.75 ± 0.38		0.82 ± 0.06	
	72 h	0.38 ± 0.52		-		2.92 ± 0.30		0.83 ± 0.07	
CSKTG	BL	0 ± 0		2.40 ± 0.62		2.95 ± 0.50		0.86 ± 0.06	
	0 h	0.13 ± 0.35		3.23 ± 0.29		2.73 ± 0.52		0.82 ± 0.05	
	24 h	0.75 ± 0.46		5.14 ± 0.31		2.73 ± 0.40		0.81 ± 0.05	
	48 h	0.38 ± 0.52		3.60 ± 0.55		2.83 ± 0.50		0.84 ± 0.06	
	72 h	0.13 ± 0.35		-		3.00 ± 0.47		0.85 ± 0.05	
Overall analysis	Huynh–Feldt	0.794		0.537		0.373		0.258	
Comparison between groups	F, p	60.667	0.000*	9.862	0.000*	3.257	0.060	2.371	0.099
Comparison between time	F, p	126.664	0.000*	1726.379	0.000*	40.448	0.000*	102.153	0.000*
P for interaction	F, p	12.602	0.000^#^	3.478	0.002^#^	5.184	0.000^#^	8.596	0.002^#^

*BL* Baseline, *0 h* Immediately after intervention; 24 h: 24 h; 48 h: 48 h; 72 h: 72 h

^*^: Two factors repeated measures ANOVA time main effect. *p* < 0.05

^#^: Interaction of two factors repeated measure ANOVA. *p* < 0.05

#### VAS value was analyzed between groups

**0 h:** The differences between CSG, KTG, and CSKTG were statistically significant compared with the CG (*P* = 0.043), (*P* = 0.015) (*P* = 0.001); **24 h:** The differences of CSG, KTG, and CSKTG were statistically significant compared with the CG (*P* < 0.001); CSKTG compared with CSG, (*P* = 0.001); CSKTG compared with KTG, (*P* = 0.028); **48 h:** The differences of CSG, KTG, and CSKTG were statistically significant compared with CG (*P* < 0.001); CSKTG compared with CSG, (*P* = 0.004); CSKTG compared with KTG, (*P* = 0.023); **72 h:** The differences of CSG, KTG, and CSKTG were statistically significant compared with CG (*P* = 0.001), (*P* < 0.001), (*P* < 0.001); CSKTG compared with CSG, (*P* = 0.036) (Table 3, Fig. 3).

**Table 3:** Comparative analysis among groups of VAS, IL-6, PT/BW, WF

		Overall		CG vs CSG	CG vs KTG	CG vs CSKTG	CSG vs KTG	CSG vs CSKTG	KTG vs CSKTG
		F	p	P	P	P	P	P	P
VAS	0 h	9.343	0.001^**^	0.043^*^	0.015^*^	0.001^**^	0.963	0.387	0.680
	24 h	33.133	0.001^**^	0.001^**^	0.001^**^	0.001^**^	0.453	0.001^**^	0.028^*^
	48 h	62.620	0.001^**^	0.001^**^	0.001^**^	0.001^**^	0.387	0.004^**^	0.023^*^
	72 h	20.311	0.001^**^	0.001^**^	0.001^**^	0.001^**^	0.244	0.036^*^	0.774
IL-6	BL	0.09	0.964						
	0 h	1.519	0.231						
	24 h	8.169	0.002**	0.392	0.301	0.042*	0.996	0.063	0.009**
	48 h	9.482	0.001**	0.266	0.045*	0.001**	0.798	0.011*	0.089
PT/BW	BL	0.302	0.823						
	0 h	1.294	0.296						
	24 h	9.447	0.001**	0.010*	0.001**	0.001**	0.710	0.570	0.996
	48 h	3.712	0.023*	0.109	0.059	0.027*	0.991	0.914	0.985
	72 h	4.761	0.008**	0.073	0.024*	0.010*	0.959	0.828	0.984
WF	BL	0.234	0.872						
	0 h	0.518	0.674						
	24 h	5.551	0.004**	0.257	0.012*	0.006**	0.476	0.315	0.990
	48 h	5.933	0.003**	0.082	0.012*	0.003**	0.839	0.535	0.952
	72 h	3.713	0.023**	0.139	0.089	0.019*	0.996	0.799	0.902

*BL* Baseline, *0 h* Immediately after intervention; 24 h: 24 h; 48 h: 48 h; 72 h: 72 h

*: *P*<0.05 for comparison between groups

**: *P*<0.01 for comparison between groups

### IL-6 value

#### The overall analysis of the IL-6 value

Shapiro Wilk test showed that the data of each group obeyed normal distribution (*P* > 0.05). There was a significant difference in the main effect between groups, F (3,21) = 243.137, *P* = 0.000, the overall grouping had a significant impact on the value of IL-6; The main effect of the time factor on the value of IL-6 in each group was statistically significant, F (3,21) = 9.862, *P* = 0.000, the overall intervention time had a significant impact on the value of IL-6. Due to the influence of group factors on IL-6 value being statistically significant, the pairwise comparison shows that the mean difference of IL-6 between CSG and CG is 1.00, *P* < 0.001; The mean difference of IL-6 between KTG and CG was 3.49, *P* < 0.001; The mean difference of IL-6 between CSKTG and CG was 2.04, *P* < 0.001; The mean difference of IL-6 between CSG and KTG was 2.49, *P* < 0.001; Compared with CSKTG, the mean difference of IL-6 was 1.04, *P* < 0.001; Compared with CSKTG, the mean difference of IL-6 was 1.46, *P* < 0.001. Due to the influence of the time factor on IL-6 value being statistically significant, the two comparisons show that the mean difference between BL and 48 h after the intervention is 0.91, *P* = 0.037; The mean difference between 0 and 48 h after the intervention was 0.58, *P* = 0.004 (Table 2).

#### IL-6 value was analyzed between groups

There was no significant difference in the average content of IL-6 between **BL** and **0 h**; **24 h:** The average content of IL-6 in CSKTG decreased by 1.68 compared with CG (*P* = 0.042); Compared with KTG, the average content of IL-6 in CSKTG decreased by 0.71 (*P* = 0.009); **48 h:** The average content of IL-6 in KTG decreased by 0.88 compared with CG (*P* = 0.045); Compared with CG, the average content of IL-6 in CSKTG decreased by 1.65 (*P* = 0.012); Compared with CSG, the average content of IL-6 in CSKTG decreased by 1.06 (*P* = 0.011) (Table 3, Fig. 4).

### PT/BW value

#### The overall analysis of PT/BW value

Shapiro Wilk test showed that the data of each group obeyed normal distribution (*P* > 0.05). The main effect of the time factor on PT/BW value in each group was statistically significant. F (4,28) = 40.448, *P* = 0.000, the overall intervention time had a significant impact on PT/BW value. Due to the influence of the time factor on PT/BW value has statistical significance, the two comparison shows that the mean difference between BL and 0 h is 0.37, *P* < 0.001; The mean difference between BL and 24 h after the intervention was 0.56, *P* < 0.001; The mean difference between BL and 48 h after the intervention was 0.36, *P* = 0.001; The mean difference between BL and 72 h after the intervention was 0.19, *P* = 0.028; The mean difference between 0 and 72 h after the intervention was 0.18, *P* = 0.040; The mean difference between 24 h after intervention and 72 h after the intervention was 0.36, *P* = 0.003; The mean difference between 48 h after intervention and 72 h after the intervention was 0.17, *P* = 0.012 (Table 2).

#### PT/BW value was analyzed between groups

**Fig. 5:**
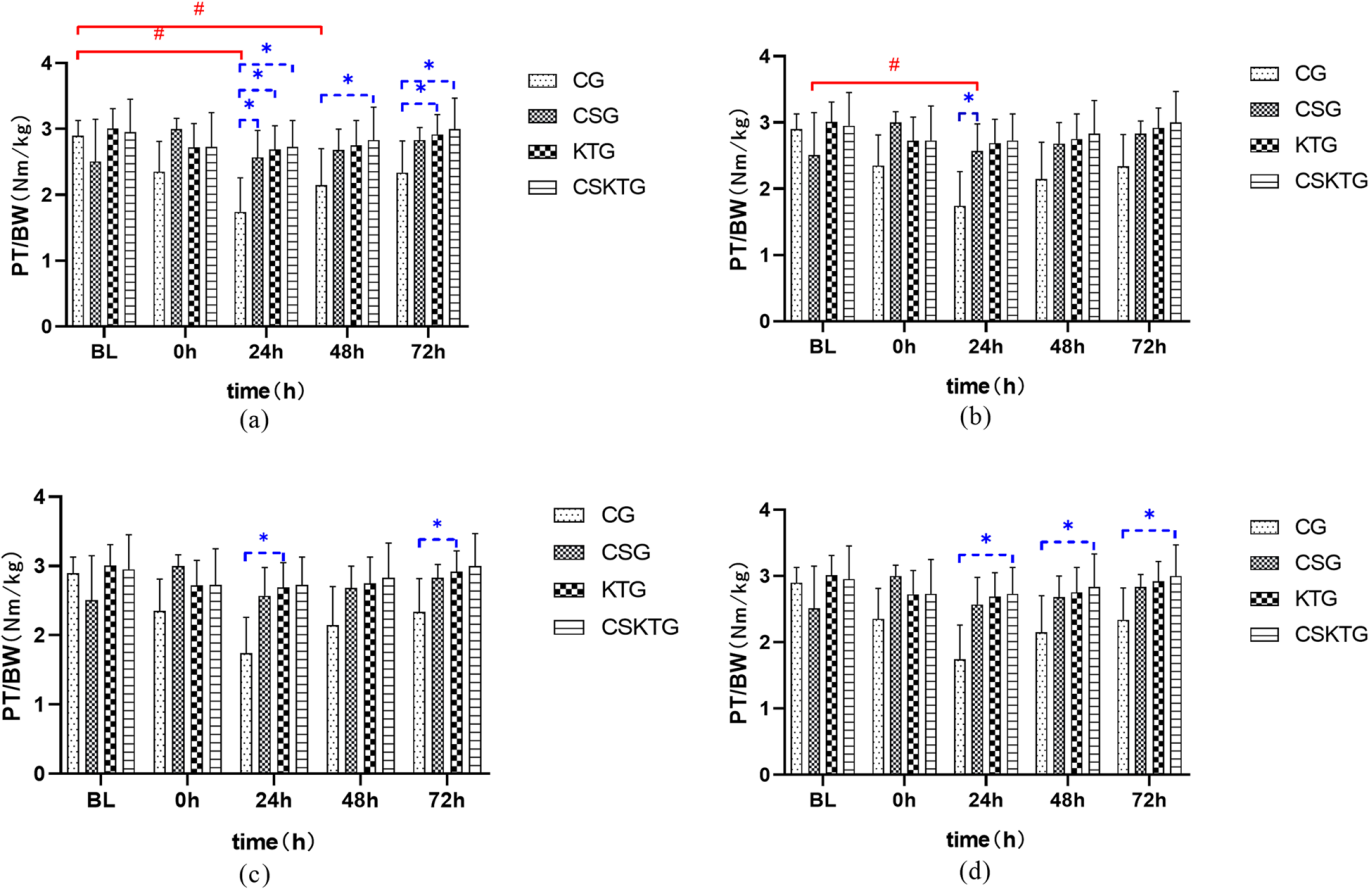
Comparison of PT/BW value between-groups and within-groups. (**a**), (**b**), (**c**) and (**d**) were the comparison of PT/BW value between-groups and within-groups in CG, CSG, KTG and CSKTG groups. *: Pairwise comparison between-groups *P*’ < 0.05. ^#^: Pairwise comparison within-groups *P*’ < 0.05

There was no significant difference in the average content of PT/BW between **BL** and **0 h**; **24 h:** Tukey test showed that compared with CG, the PT/BW of CSG decreased by 0.72 on average (*P* = 0.010); Compared with CG, PT/BW of KTG decreased by 0.94 on average (*P* = 0.001); Compared with CG, the PT/BW of CSKTG decreased by 0.99 on average (*P* < 0.001); **48 h:** Tukey test showed that the PT/BW of CSKTG increased by 0.67 on average compared with CG (*P* = 0.027); Compared with KTG, the PT/BW of CSKTG increased by 0.71 on average (*P* = 0.009); **72 h:** Tukey test showed that PT/BW of KTG increased by 0.58 on average compared with CG (*P* = 0.024); Compared with CG, the PT/BW of CSKTG increased by 0.65 on average (*P* = 0.010) (Table 3, Fig. 5).

### WF value

#### The overall analysis of the WF value

Shapiro Wilk test showed that the data of each group obeyed normal distribution (*P* > 0.05). There was no significant difference in the main effect between groups, F (3,21) = 2.142, *P* = 0.099, the overall grouping had no significant effect on the WF value; The main effect of the time factor on the TW value in each group was statistically significant, F (4,28) = 18.879, *P* < 0.001, the overall intervention time had a significant impact on WF value. Due to the influence of the time factor on WF time being statistically significant, the two comparisons show that the mean difference between BL and 0 h is 0.06, *P* < 0.001; The mean difference between BL and 24 h after the intervention was 0.11, *P* < 0.001; The difference between the mean of 48 h after intervention and BL < 0.001 was *P* < 0.001; The mean difference between BL and 72 h after the intervention was 0.05, *P* < 0.001; The mean difference between 0 and 24 h after the intervention was 0.05, *P* < 0.001; The mean difference between 0 and 48 h after the intervention was 0.02, *P* = 0.042; The mean difference between 24 h after intervention and 48 h after the intervention was 0.03, *P* = 0.004; The mean difference between 24 h after intervention and 72 h after the intervention was 0.05, *P* = 0.002; The mean difference between 48 h after intervention and 72 h after the intervention was 0.02, *P* = 0.005 (Table 2).

#### WF value were analyzed between groups

**Fig. 6:**
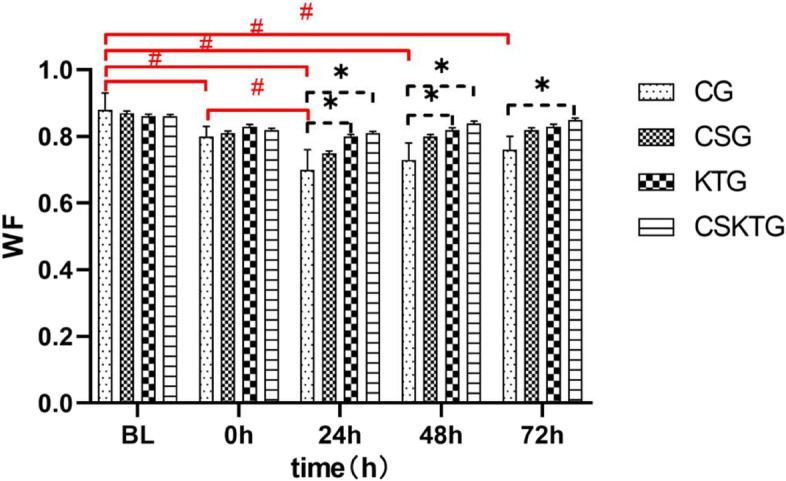
Comparison of WF value between-groups and within-groups. *: Pairwise comparison between-groups *P*’ < 0.05. ^#^: Pairwise comparison within-groups *P*’ < 0.05

There was no significant difference in WF between **BL** and **0 h** intervention groups. **24 h:** The mean difference in WF value between KTG and CG was 0.10 (*P* = 0.012); The mean difference in WF value between CSKTG and CG was 0.11, (*P* = 0.006); **48 h:** The mean difference of WF value between KTG and CG was 0.10 (*P* = 0.012); The mean difference of WF value between CSKTG and CG was 0.11, (*P* = 0.003); **72 h:** The mean difference of WF value between CSKTG and CG was 0.09 (*P* = 0.019) (Table 3, Fig. 6).

## Discussion

The purpose of this study is to reveal the effect of KT and CS on DOMS. The main hypothesis was that KT combined with CS can alleviate DOMS by reducing inflammation, muscle damage, and fatigue. Through our research, this hypothesis was partially supported. Our main findings were shown as follows: (1) Compared with the control group, after using KT and CS, the pain score, fatigue index and occurrence degree of DOMS of lower limb muscles were significantly reduced after exercise immediately; (2) Compared with baseline, IL-6 and PT/BW value recovered faster after KT and CS were used together.

Pain is a multi-dimensional experience and a prominent feature of many musculoskeletal diseases. Accurate and reliable measurement is the premise of effective treatment. Although pain is subjective, it is a highly relevant complaint. Visual Analog Scale (VAS) is a quantitative assessment tool for pain, which can guide the determination of the impact of pain on the body [38]. Pain is mainly caused by harmful stimuli. Although the pain will cause discomfort, it also can stimulate a defensive protective response and thus serve as a warning of injury [39, 40]. It can be seen from the result analysis that compared with the CG, after using CS, KT, or CS combined with KT, the occurrence degree of DOMS and pain decreased and showed a decreasing trend. CG has the most obvious pain in 24 h after exercise, and the pain change range is the largest, indicating that DOMS occurs most obviously without external protection. CS combined with KT is superior to KT or CS alone in alleviating pain. KT can increase muscle strength by transmitting the tension that promotes alternating fascia movement, promotes muscle activity, and improves muscle strength at the same time [41]. Muscle soreness limits muscle performance. However, KT can reduce muscle soreness, which is consistent with previous studies [42]. The hypothesis of the KT folding effect can explain the results of this study to some extent. The folds of the adhesive tape lift the skin of the local tissues and increase the space below [43]. The blood and lymph circulation accelerate to enter the local tissues so that more anti-inflammatory factors can penetrate the lesions and accelerate the inflammatory reaction. In addition, studies have found that inflammation and inflammatory reactions can cause pain, and IL-6 can inhibit proinflammatory cytokines and mitigate neuropathic pain.

Zeynep et al. concluded that KT intervention can reduce the muscle soreness of the quadriceps femoris in fifty-four nonathletic volunteers [44]. Rafael et al. found that KT significantly attenuated the effects of acute lumbar muscle soreness experienced by athletes during cross-country skiing practice [45]. Therefore, this study speculates that KT attached to the thigh muscle acts as a continuous analgesic stimulation, which can increase the participation of skin receptors and can attribute to the interaction between skin receptors and the pain pathway. CS can reduce the delayed onset of upper and lower limb muscle soreness (DOMS), relieve muscle swing, improve joint mobility, reduce oxygen consumption during sub-extreme exercise, change local blood flow and clearance of protein or metabolites, alleviate swelling and muscle soreness in the recovery period [29]. The pain of DOMS will not be weakened as the increase of transmission distance, which indicates that the whole muscle tissue produces the same pain, the key painful parts are not obvious. The reason why CS combined with KT has a better effect on DOMS prevention and pain mitigation is that they have a superposition effect, which can reduce the degree of pain and accelerate the recovery of pain.

The development of muscle damage caused by DOMS is a process of increasing pain and body load. Therefore, participants should pay attention to the recovery process of physical fatigue and DOMS. After the occurrence of DOMS, the subjects were asked to rest for a while and reduce the amount of exercise when the pain reaches the maximum value to avoid the aggravation of pain and the development of tissue injury into muscle damage. It can be seen from the increase of VAS in different intervention groups 24 h after exercise. After using CS, KT, or CS combined with KT, the occurrence degree of DOMS and pain showed a decreasing trend, indicating that these additional measures played a protective role in DOMS and prevented the occurrence and development of DOMS.

IL-6 is a cytokine related to exercise. It is involved in a complex series of biological reactions in the body, often playing a role in inflammation, immune response, and bone metabolism. At the same time, it is also a strong inflammatory response factor that causes pain and can be used as one of the important outcomes of the inflammatory response [46–48]. The mechanism of inducing IL-6 production is different in muscle contraction and muscle tissue injury and inflammation. After muscle damage exercise, muscle tissue injury caused by high-intensity eccentric contraction will promote the infiltration of inflammatory cells [49–51]. The current research shows that the contracted skeletal muscle of the body produces and releases a large amount of IL-6 during exercise [52]. Takuji et al.'s research on the role of neutrophil dynamics and function in exercise-induced muscle injury and delayed muscle soreness shows that plasma IL-6 is significantly correlated with muscle pain (VAS score), and peripheral blood neutrophil count and plasma IL-6 concentration significantly correlated with indirect muscle damage markers [53]. Zhao et al. found in the comparative study on the therapeutic effect of different cold treatment schemes on delayed muscle soreness that IL-6 changes first after skeletal muscle micro-injury. IL-6 indicates delayed muscle soreness and should be sensitive to prostaglandin-2. IL-6 is one of the most sensitive and early outcomes related to muscle micro-injury [54].

Skeletal muscle damage is the main cause of IL-6 release caused by exercise. It is also related to exercise stress, exercise intensity, heart rate during exercise, adrenaline, and so on [55, 56], the results of this study are the same. The use of CS, KT, or CS combined with KT has a great impact on the level of IL-6 after vigorous exercise. At 24 h after the intervention, the content of IL-6 reached a peak. There was a significant difference between CS combined with KT and single-use of KT or CG, and there was no significant difference between CS combined with KT and CS. It is suggested that the effect of CS on reducing inflammatory factors may be slightly better than KT. We speculate the possible reason is that CS can improve muscle oxygen supply, alleviate DOMS, reduce blood lactic acid concentration, and finally promote the clearance of metabolic end products, promote the rapid reduction of inflammatory factors, and accelerate the regression of fatigue after heavy exercise. Due to the large differences between different intervention groups, the impact of these interventions on IL-6 levels should also be carefully considered, and further research should be conducted to provide additional evidence.

A large number of studies have shown that peak torque is the expression of the maximum torque in the process of joint flexion and extension, the embodiment of muscle strength, and has good repeatability. It is regarded as the gold standard and reference value of the isokinetic test, while relative peak torque (PT/BW) is the percentage of peak torque in body weight, excluding the influencing factors of body weight, which can be used to compare the torque between different individuals. The intergroup analysis found that compared with the CG, the improvement of PT/BW by using CS, KT, or CS combined with KT at 24 h after the intervention was statistically significant. It shows that after the production of DOMS, CS and KT can inhibit DOMS, to improve PT/BW. The intragroup analysis found that the PT/BW of CG decreased first and then increased after modeling. At 72 h, the upward trend still did not return to the mean value before modeling. The reason for the above results may be that once DOMS is produced, it will spread to the whole muscle participating in the exercise. The degree of muscle tissue damage is directly proportional to the recovery time and inversely proportional to the exercise ability. The more serious the injury is and the longer the recovery time is, the weaker the exercise ability is, resulting in such a changing trend of PT/BW [57].

WF is the main index to reflect the strength and endurance of lower limb joint muscles. There is no single cause of muscle fatigue. The main mechanism is specific to those processes under pressure during fatigue exercise. This concept is similar to the principle of specificity [58]. How does fatigue affect muscle function? By definition, once a muscle's maximum strength or explosive power begins to decline, it begins to feel tired. When the task involves maintaining maximum shrinkage, the performance decline is parallel to the increase in fatigue. However, when the exercise task requires submaximal contraction, the onset of fatigue may not be related to the termination of the exercise task. Since most activities of daily living involve submaximal forces, the occurrence of fatigue may not limit an individual's ability to perform motor tasks. In addition, the failure to perform motor tasks may be caused by the fatigue of the main muscles involved in the task [59]. According to the test results, under the protection of KT, the occurrence of DOMS was delayed. For CG, after the occurrence of DOMS, fatigue also exists, and the fatigue will gradually increase with the aggravation of DOMS.

According to research and analysis, when CS is used, perceived fatigue is significantly reduced. Research shows that wearing CS within 24 h after high-intensity resistance training can significantly reduce perceived fatigue [60]. The beneficial effects of KT and Cs on DOMS and perceived fatigue may be that after pressure is applied to the limbs, the space for swelling and edema is reduced, resulting in less change in osmotic pressure, which will reduce the diffusion of liquid in the interstitial space and better venous reflux [42]. It can be seen that KT and CS have better recovery abilities for DOMS fatigue feeling, and have a wide application space and prospect.

### Limitations and advice

There are several limitations to the present study. First of all, this study only tested the blood outcomes at 48 h but did not analyze the blood outcomes for 72 h, which may also have a certain impact on the reliability of the conclusion. Secondly, due to the difficulty in controlling the amplitude and the height of the jump during the DOMS modeling process, the effect after the jump may be inconsistent and the results may be biased. Thirdly, the sample size of this study is relatively small. To increase the universality, it is necessary to expand the research participants, increase the sample size, and observe more deeply the short-term and long-term effects and action mechanisms of KT and CS application schemes.

The mechanism of action of KT is to affect the tension force of the skin and promote blood circulation through the density difference of the patch when sticking. Reasonable use can enhance the contractility of damaged muscles, eliminate local pain, reduce muscle overstretching, and reduce muscle fatigue and spasms. The function of CS is to wrap the muscles, contract and gather the muscle groups, and make the muscles more powerful. It can provide good support for muscles, reduce muscle shaking, and achieve local protection, provide stability. The method adopted by CSKTG is to use CS combined with KT. Since both CS and KT are elastic, KT plays a role in direct contact with the skin. At this time, the position of CS is applied on top of KT. Since the two methods are superimposed, because of the CS cover, KT may it cannot play a normal role. On the other hand, from the experimental results, it can be seen that CS combined with KT has a better effect on preventing DOMS, and the superimposed effect of the two is better than that of CS or KT alone. It can be explained that there are similarities in the working principles of KT and CS. At the same time, KT may make CS more fit within a reasonable range and may enhance the stability and effectiveness of CS. Therefore, whether the application of CS on KT will limit the functionality of KT needs further experiments to verify.

In this study, male participants without systematic training were selected, in the later research, female participants can be studied to test the reliability of the results; It is suggested that KT and CS can be carried out after DOMS caused by different sports in the future. In addition, the mechanism of KT and CS to prevent DOMS muscle damage needs to be further elucidated.

## Conclusions

This study indicated that KT can significantly reduce DOMS pain, and KT has a better recovery effect on DOMS than CS. KT combined with CS can mitigate DOMS pain, accelerates isokinetic muscle strength recovery, and shorten the recovery time of the body after DOMS, it also accelerates recovery from Peak Torque/Body Weight and Work Fatigue. KT combined with CS has a more obvious recovery effect on DOMS.

## Data Availability

The datasets used and analysed in this study are available from the corresponding author on reasonable request.
